# Perspective: leveraging review protocols to advance implementation science in support of older adults’ health equity

**DOI:** 10.3389/fpubh.2024.1457591

**Published:** 2024-10-11

**Authors:** Juanita-Dawne Bacsu, Avery Pottle, Florriann Fehr, Megan Funk, Matthew Lee Smith

**Affiliations:** ^1^School of Nursing, Thompson Rivers University, Kamloops, BC, Canada; ^2^Department of Medicine, Queen's University, Kingston, ON, Canada; ^3^Department of Health Behavior, Center for Community Health and Aging, Center for Health Equity and Evaluation Research, School of Public Health, Texas A&M University, College Station, TX, United States

**Keywords:** health equity, review, protocol, implementation science, older adults, aging

## Abstract

Globally, there has been an increasing call for dissemination and implementation science to improve the health equity of older adults. However, improving the health equity of older adults requires methods that allow for an in-depth understanding of the existing research. While the value of review articles is widely recognized, the value of publishing review protocols is an emerging paradigm with great potential to advance implementation science and aging research. In this article, we propose that prior to review articles, review protocols are necessary as they provide a clear and comprehensive search strategy, which greatly enhances the quality and rigor of reviews. More specifically, we argue that review protocols are indispensable because they support: (1) a clearly defined and comprehensive search methodology; (2) reviewer feedback to mitigate potential issues; and (3) registration to reduce risk of research replication. Additionally, we propose three strategies to accelerate review protocols in implementation science to support older adults’ health equity.

## 1 Introduction

Urgent action is needed to improve the health equity of older adults. By 2030, 1-in-6 people worldwide will be ages 60 years and older (1). Health inequity refers to unfair, unjust, and avoidable differences in health outcomes at the population level (2). The World Health Organization and the United Nations Decade of Healthy Ageing initiative notes that several determinants fuel health inequity among older adults such as socio-economic status, physical and social environment, gender, access to health and support services, and ageism (3). Despite this knowledge, health inequities impacting older adults persist (4, 5). For example, ageism continues to decrease older adults’ lifespan, reduces mental and physical health, delays recovery from injury, hastens cognitive decline, and has been linked to decreased longevity (6).

Globally, there has been an increasing call for dissemination and implementation science to improve the health equity of marginalized populations (7, 8). Study methods are now being investigated to advance health equity in implementation science (9). However, improving the health equity of older adults requires methods that allow for an in-depth understanding of the existing research within the field.

In this context, we contend that review articles (such as scoping and systematic reviews) are critical to advancing implementation science, especially in relation to improving the health equity of older adults. Review articles identify theoretical models, reveal knowledge gaps, disclose research contradictions, synthesize extant knowledge, and provide a “one-stop-shop” to map evidence-informed research (10). Ketchen and Craighead (11) note that a high-quality review identifies advances within a research topic and provides a strong foundation to excel future research and inquiry. Consequently, understanding evidence-informed knowledge is essential to implementing research into action to improve older adults’ health equity at the individual, community, and policy levels. Review articles specifically pertaining to implementation science are particularly important because they synthesize evidence about various elements of planning, disseminating, implementing, and evaluating interventions and strategies that can guide efficiency among researchers, practitioners, and policy makers to optimize strategies pertaining to the reach, adoption, effectiveness, and maintenance of their efforts.

While the value of review articles is widely recognized, the value of publishing review protocols is an emerging paradigm with great potential to advance implementation science and aging research (12–14). In this article, we propose that prior to review articles, review protocols are necessary as they provide a clear and comprehensive search strategy, which greatly enhances the quality and rigor of reviews. More specifically, we argue that review protocols are invaluable because they support: (1) a clearly defined and comprehensive search methodology; (2) reviewer feedback to mitigate potential issues; and (3) registration to reduce risk of research replication. Additionally, we propose three strategies to accelerate review protocols in implementation science to support older adults’ health equity.

### 1.1 Supports a clearly defined and comprehensive search methodology

Review protocols are vital to providing a clearly defined and comprehensive search methodology thereby enhancing rigor of the review. Specifically, experts from the Joanna Briggs Institute (15) note that protocols enable in-depth planning of the reviews, leading to clear research questions, transparent and reproducible work, and reduced selection bias and subjectivity. Moreover, review protocols enable researchers to collaborate with expert librarians to create a comprehensive search strategy, including search terms, inclusion/exclusion criteria, timelines, and credible databases. This comprehensive strategy ensures trustworthiness through transparency and reproducibility of the results, along with limiting selection bias by preventing late-stage inclusion decisions (16). Cochrane emphasizes the importance of a transparent review protocol, which pre-defines the review’s objectives, methods, and reporting, with any deviations from the protocol requiring a clear explanation (17). Utilizing a well-defined review protocol can help researchers improve review clarity, reproducibility, and transparency, along with reducing bias and subjectivity.

### 1.2 Allows for reviewer feedback to identify potential issues and mitigation strategies

Providing a review protocol also allows reviewers to identify potential issues and oversights in the methodology of the search, mitigating biases and identifying gaps in the literature that may have otherwise been overlooked. A well-defined framework allows reviewers to quickly identify errors, whether it be the rationale for the research question(s), the search terms, the inclusion/exclusion criteria, or the breadth and diversity of the studies to be included. For example, a recent review examining stigma toward older adults living with dementia was greatly enhanced by the protocol reviewers’ feedback suggesting a more nuanced examination of the different types of stigmas (self, public, and societal stigma) on health outcomes (18, 19).

### 1.3 Promotes registration to reduce the risk of research replication

Registries enable researchers to explore on-going reviews within the field of aging and health equity, promoting collaboration and reducing replication and publication bias. Public registries such as Open Science Framework and the Cochrane registration facilitate collaboration among researchers by providing clear review protocols that can serve as the foundation for subsequent projects. Additionally, registries prevent unintentional duplication by creating a universal source to access on-going research before beginning a review. Registries also address publication bias because researchers maintain access to the content within registries, despite a significant proportion of registered studies not advancing to peer-review publication. Accordingly, registries facilitate collaboration among researchers, reduce unintended replication, and minimize publication bias by increasing the accessibility of unpublished work.

## 2 Discussion: three strategies to advance review protocols

Review protocols are essential to providing a clearly defined and comprehensive search methodology for reviews, especially for complex topics such as implementation science and addressing the health inequity of older adults. However, there continues to be insufficient attention paid to the need for review protocols. We recommend three strategies to fuel the advancement of review protocols and subsequent reviews in implementation science: (1) enhance education and awareness; (2) implement protocol review requirements; (3) and increase journal publication options.

### 2.1 Enhance education and awareness about review protocols

There is a crucial need for increased education and knowledge about review protocols. This strategy could be achieved through enhanced education and advertising of existing review registries such as JBI, Cochrane, Campbell Collaboration, PROSPERO, and Open Science Framework. Although various registries exist, some reviews remain unregistered due to lack of protocol knowledge and registration options. Strategies to enhance awareness about review protocols and registries may include educational workshops, webinars, and guest speakers on protocol reviews and registries targeting graduate students and faculty members. JBI notes that review protocols must be viewed as an invaluable and indispensable component of the review process that can prevent last-minute decision making and arbitrary reporting (20).

### 2.2 Implement protocol review requirements

Another strategy to advance review protocols is to implement protocol review requirements. For example, journals are now beginning to require reviews to be registered prior to the review. However, journals could also require that review protocols be published as prerequisites to the review to help ensure rigor and high-quality reviews. Moreover, grant funding agencies could implement review protocol requirements as preconditions to grant funding for reviews. Additionally, universities could teach their researchers and graduates students about review protocols as part of the research process to ensure proper planning. Moreover, institutional incentives to encourage review protocols could include internal funding opportunities or institutional memberships such as to the Joanna Briggs Institute to support protocol development.

### 2.3 Increase journal publication options

Additional journals and dissemination venues are needed to publish review protocols. Currently, only a handful of journal publishers will accept review protocols such as BMC journals’ *Systematic Reviews*, *BioMed Central Protocols*, *BMJ Open*, and *JMIR Protocols*. Protocols can also be published through other means such as *Cochrane Reviews* (published by Wiley) and the *JBI Evidence Synthesis* that publishes review protocols. However, some dissemination venues such as JBI requires institutional membership which creates barriers to protocol publication. Solutions to promote review protocols could be the creation of new journals with relevant aims and scopes and/or the introduction of new article types specific to review protocols.

## 3 Conclusion

Review protocols are essential to developing high-quality review articles necessary to advance implementation science, especially for complex topics such as addressing the health equity of older adults. Protocols provide a strong foundation for a review by clearly defining the search parameters and search methodology. However, there continues to be insufficient recognition surrounding the importance of review protocols. Moving forward, review protocols and subsequent review articles must be recognized as valuable methods to accelerate implementation science, especially for improving complex issues. Understanding evidence-informed knowledge is crucial to implementing research into action to improve older adults’ health equity at the individual, community, and policy levels.
